# Coronary angiography video segmentation method for assisting cardiovascular disease interventional treatment

**DOI:** 10.1186/s12880-020-00460-9

**Published:** 2020-06-16

**Authors:** Dongxue Liang, Jing Qiu, Lu Wang, Xiaolei Yin, Junhui Xing, Zhiyun Yang, Jiangzeng Dong, Zhaoyuan Ma

**Affiliations:** 1 The Future Laboratory, Tsinghua University, Chengfu Road, Beijing, China; 2 Center for Cardiology, Beijing Anzhen Hospital, Capital Medical University, Anzhen Road, Beijing, 100029 China; 3 The First Affiliated Hospital of Zhengzhou University, Zhengzhou, 450052, China, 1 Jianshe East Road, Erqi District, Zhengzhou, 450052 China

**Keywords:** Coronary angiography, Medical assistance, Video segmentation, Medical imaging, Medical assistance

## Abstract

**Background:**

Coronary heart disease is one of the diseases with the highest mortality rate. Due to the important position of cardiovascular disease prevention and diagnosis in the medical field, the segmentation of cardiovascular images has gradually become a research hotspot. How to segment accurate blood vessels from coronary angiography videos to assist doctors in making accurate analysis has become the goal of our research.

**Method:**

Based on the U-net architecture, we use a context-based convolutional network for capturing more information of the vessel in the video. The proposed method includes three modules: the sequence encoder module, the sequence decoder module, and the sequence filter module. The high-level information of the feature is extracted in the encoder module. Multi-kernel pooling layers suitable for the extraction of blood vessels are added before the decoder module. In the filter block, we add a simple temporal filter to reducing inter-frame flickers.

**Results:**

The performance comparison with other method shows that our work can achieve 0.8739 in *Sen*, 0.9895 in *Acc*. From the performance of the results, the accuracy of our method is significantly improved. The performance benefit from the algorithm architecture and our enlarged dataset.

**Conclusion:**

Compared with previous methods that only focus on single image analysis, our method can obtain more coronary information through image sequences. In future work, we will extend the network to 3D networks.

## Background

Cardiovascular disease, especially coronary sclerotic heart disease, also known as coronary heart disease, is one of the diseases with the highest mortality rate. Traditionally, doctors diagnose these cardiovascular diseases by directly observing angiographic images with their eyes. According to experience, they make a qualitative judgment on the patient’s condition. This diagnostic method is greatly affected by human factors and lacks accuracy and objectivity. With the widespread application of image segmentation technology in the field of medical image analysis and processing, and the increasing emphasis on the prevention, diagnosis, and treatment of cardiovascular disease, the segmentation of coronary angiography images and videos has gradually become a research hotspot. The segmentation and extraction of coronary blood vessels are also gradually being applied, in practice such as assisted diagnose of the disease, precise location of lesions, quantitative analysis of vascular tissue, and research on three-dimensional reconstruction of coronary arteries.

Due to the particularity of medical images, there have been some difficulties in the processing and analysis of the images. The analysis of angiograms has the following difficulties: first, the shape of the blood vessels in the video is complex and easily deformed. Blood vessels have a tubular curved structure, and some blood vessels can block, cover or entangle with each other, which brings some difficulties to image processing. Second, the density and diameter of blood vessels vary. With the extension of the blood vessels, the blood vessels gradually become thinner, and there is vascular stenosis caused by the blockage. This causes the contrast and resolution of the small blood vessels in the contrast image to be very low and difficult to process. Third, the background noise in the image is relatively high. In coronary angiography images, besides blood vessels, there are different tissues such as the chest, lungs, and ribs. The shape or grayness of some tissues is similar to that of the blood vessels, which makes it difficult to extract blood vessels.

So far, many methods have been used to segment blood vessels. These methods maybe classified as follows: pattern recognition, model-based tracking, propagation, and artificial intelligence-based methods [1–5]. Most of the vessel detection methods are performed in the spatial domain of the original image such as single-scale top-hat operator [6], hit-or-miss transform [7], and Gaussian matched filter [8]. Poli [9] also proposed an efficient method based on linear filters, which used a shifted Gaussian kernel as it is more sensitive to vessels with different orientations and radius. An adaptive tracking method was also presented to extract vessels from the X-ray angiograms [10]. Model-based approaches for medical image segmentation were also applied. In [11], the author proposed a snake model to extract the vessels by deforming the spline to minimize energy functions. All these previous methods for vessel segmentation are limited by at least one of the following disadvantages: they either were unable to suppress sudden noise, could not detect vessels in a wide range from a fixed scale image, or needed heavy computation for the vessel segmentation.

Nowadays, the learning-based methods were very popular in segmenting medical images. An unsupervised learning-based method was proposed by Aganj [12] to segment the X-ray and MRI images. Tong [13] also proposed a multi-organ segmentation method by combing dictionary learning and sparse coding. There was also some work [14, 15] using the pixel-level classification method to get the ROI of the medical image by using the pre-trained data. The main drawback of these methods is that it is difficult to design the representative features for different applications, and if we change the kind of the input image, it is hard to capture the features. With the development of the deep learning method, the convolutional neural network played an important role in medical image analysis, such as [16, 17]. Different from the traditional classification method, deep learning methods learn the features automatically. For the medical image segmentation, most of the earlier deep learning-based methods use the image patches and sliding window block like [18]. But this kind of method will have a huge amount of computation caused by sliding the window block and ignore the global feature at the same time. In 2015, U-net [19] was proposed for medical image segmentation and achieve a good result. After that many different methods based on U-net architecture had been proposed. M-net [20] added a multi-scale input image and deep supervision to the original U-net architecture. Also, some new modules were proposed to replace some blocks in the U-net architecture to enhance the feature learning ability. Gibson et al.[21] proposed a dense connection in the encoder block to do the organ segmentation task on CT images. Also, to improve the segmentation performance, Zhao et al.[22] introduce a modified U-net by adding a spacial pyramid pooling.

The U-net and its modification methods have a common drawback which is the continuous pooling and convolution striding will lose the feature resolution, and this is a big limitation for segmenting the vessels witch require small spatial information. Especially, in medical video analysis, the continuous segmentation between frames needs more detail information of the segmentation results. With the previous discussion and reference the work of Gu et al.[23], in this paper, we focus on a new application of the segmentation method used for coronary angiography video. Similar to [23], the method combines with three main modules: the video encoder module, the video decoder module, and the video filter module. In the encoder module, to achieve more high-level semantic features, a dense atrous convolution (DAC) block and a residual multi-kernel pooling (RMP) block are added. After the normal decoder module, we add a video filter block to get the smooth video sequences.

The main contributions of our work are summarized as follows:

– A novel method to extract vessel from the coronary video.

– A new application of the segmentation algorithm used in the medical assistance field.

– A new dataset used for coronary angiography video segmentation.

## Methods

**Fig. 1:**
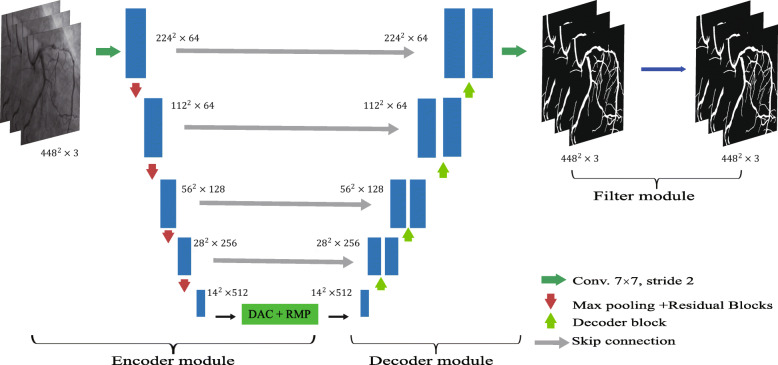
Framework for video segementation

Figure 1 shows an overview of the proposed method. The method uses the coronary angiography video sequences as input, and outputs are the segmented sequences. The proposed method consists of three main parts: the sequence encoder module, the sequence decoder module, and the sequences filter module.

### Encoder module

We use the U-net as the basic architecture for the proposed method. In this architecture, the original encoder replaced with the pre-trained ResNet-34, as ResNet can avoid gradient disappearance and accelerate network convergence. In the ResNet-34, we keep the first four feature extracting blocks only. We use the modified U-net with pre-trained ResNet as the backbone method.

**Fig. 2:**
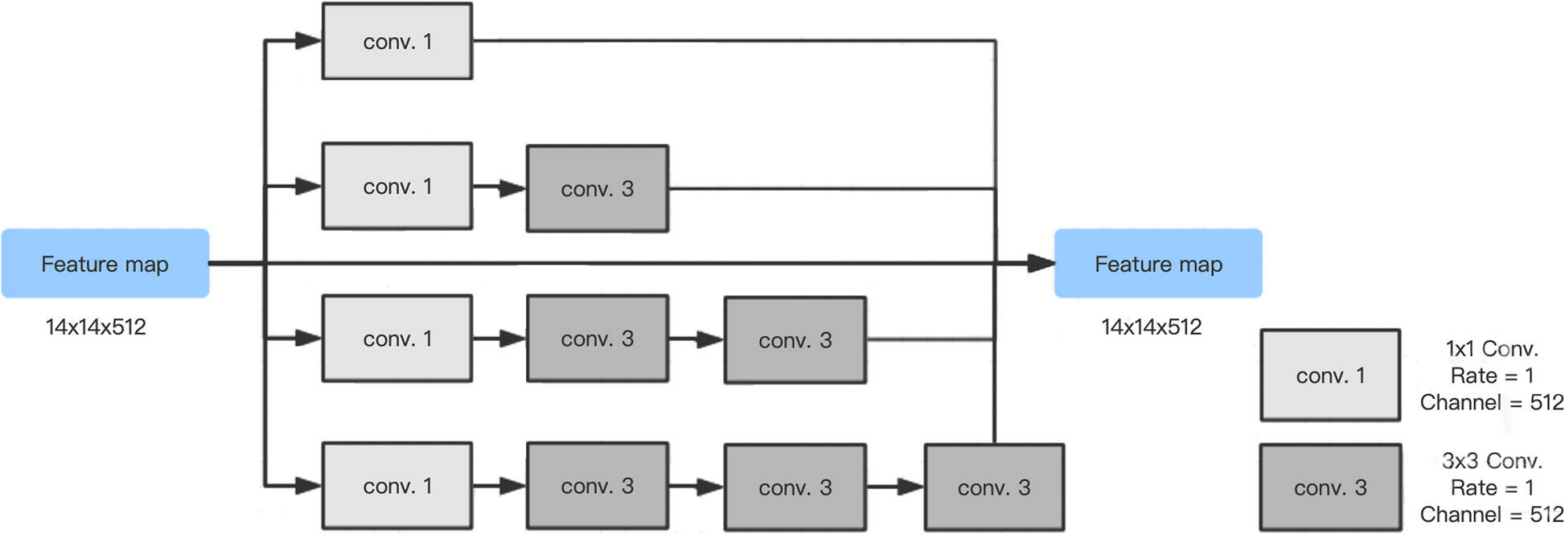
Dense atrous convolution module

The context extractor consists of the DAC (Dense Atrous Convolution) block and the RMP (Residual Multi-kernel pooling) block [23], which can extract more high-level features. Based on the Inception-ResNet-V2 block [24] and atrous convolution [25], high-level features can be encoded by the dense atrous convolution (DAC) block. As shown in Fig. 2, we use the same four cascade branches with the gradual increment of the number of atrous convolution in the DAC block, this block employs different receptive fields. The convolution of a large reception field could extract and generate more abstract features of large objects, while the convolution of a small reception field is better for small objects. By combining the atrous convolution of different atrous rates, the DAC block can extract features for objects with various sizes. A residual multi-kernel pooling (RMP) is also adopted in this method, which works on a large variety of object sizes in a medical image, like coronary angiography with different sizes of vessels. The RMP extracts the feature with four different sizes of poolings: 2 ×2, 3 ×3, 5 ×5 and 6 ×6. After every pooling step, a 1 ×1 convolution is used to reduce the dimension of the computational cost. In the end, by bilinear interpolation, we do the upsampling to get the final features.

### Decoder module

Using the decoder module, we can recover the high-level features extracted from the feature extraction encoder module. Just like U-net, as normal striding convolution and continuous pooling operations will lead to loss of information, we can skip the connection and transfer some detailed information directly from the encoder to the decoder. The upscaling and deconvolution are two common operations of the decoder in U-net. The upscaling operation increases the image size by linear interpolation, while the deconvolution uses a convolution operation to enlarge the image. Here, it mainly includes 1 ×1 convolution, 3 ×3 transposed convolution, and 1 ×1 convolution consecutively. The deconvolution operation can adaptively learn the mapping to recover features using more detailed information. Therefore, in our method, deconvolution was selected to recover the high-resolution features in the decoder. The output of the final feature recovery decoder module is a mask of the same size as the original input image.

### Video filter module

**Fig. 3:**
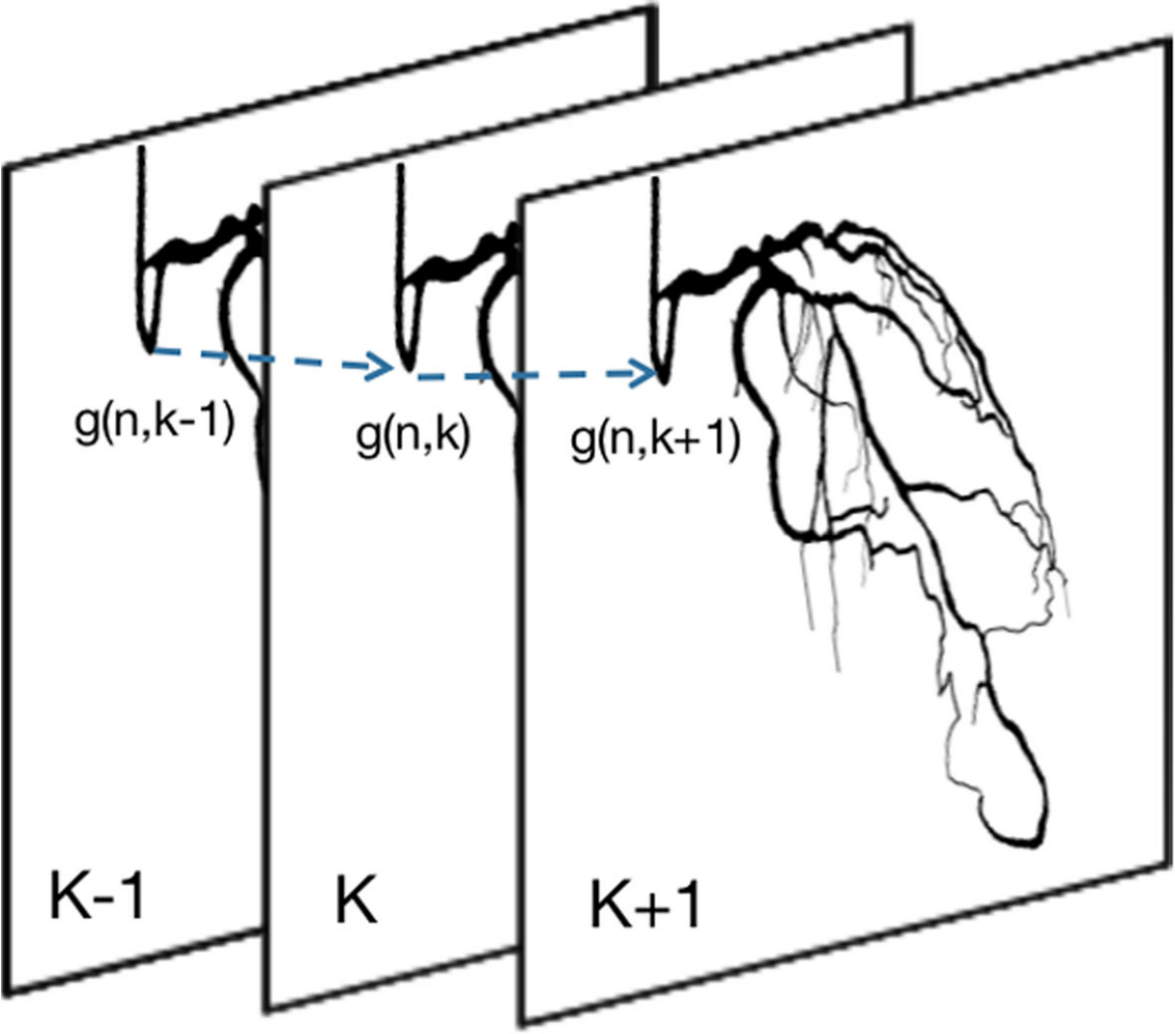
Video filter of the image element (**n**,k)

We observe that there exists some confusion between frames in the output mask from the decoder. Since we only do the segmentation in a one-shot manner without considering temporal information in other frames. To improve the segmentation results and reduce computational complexity, we use a simple temporal filter to carry out a weighted averaging of successive frames [26]. As shown in Eq.(1),

1 $$ \hat{f}(\mathbf{n}, k)=\sum_{l=-K}^{K} h(l) g(\mathbf{n}, k-l)  $$

where *g*(**n**,*k*) is the recorded image sequence, *k* is the number of the sequence. **n**= (*n*1,*n*2) refers to the spatial coordinates. *h*(*l*) are the temporal filter coefficients used to weight 2*K*+1 consecutive frames. In case the frames are considered equally important we have *h*(*l*)=1/(2*K*+1). The motion artifacts can greatly be reduced by operating the filter along with the image elements that lie on the same motion trajectory [27], as shown in Fig 3.

## Results

### Data

**Fig. 4:**
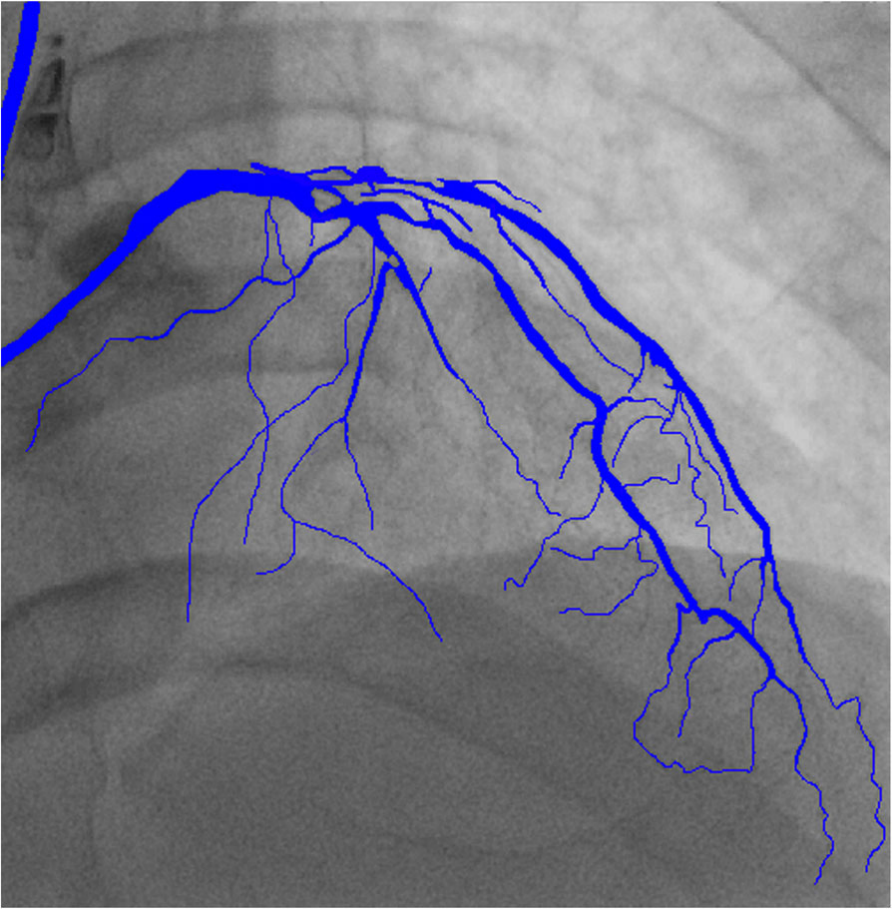
Annotated image

In our work, we choose two parts of the dataset for training, one is the public DRIVE [28] dataset which is widely used for vessel detection, and the other dataset comes from the angiography video of coronary interventional surgery. 170 video clips contain clear blood vessels with contrast agents were annotated manually by medical students. We record hundreds of videos of interventional surgeries, clip the videos which have contrast agents, then do interframe sampling about every three frames, finally we get 4904 annotated angiography images, as shown in Fig. 4. The annotated images are divided into two sets by selecting 4354 images for the train set and 550 images for the validation set during the training.

### Train

**Fig. 5:**
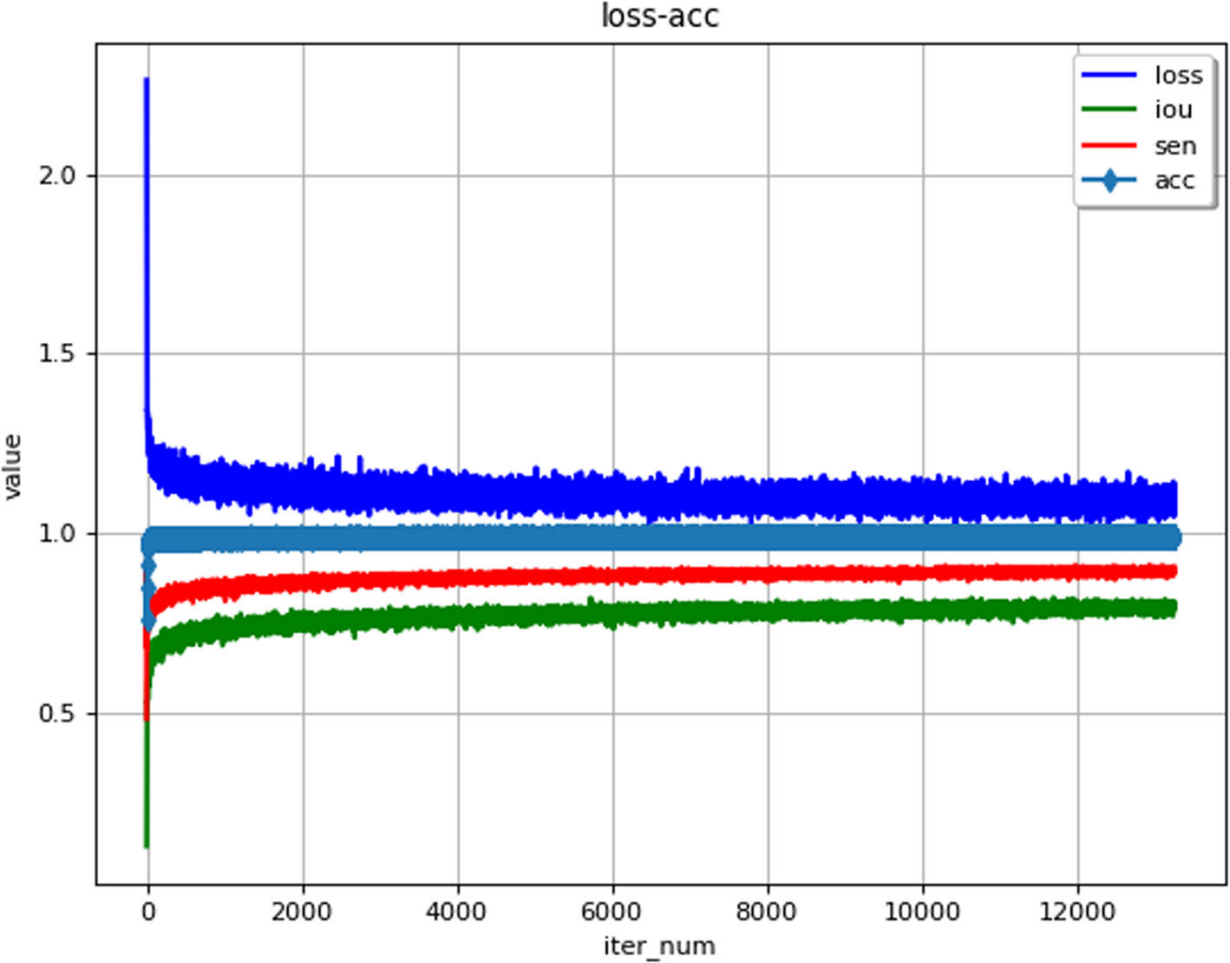
Learning curves

**loss function** This paper uses the end-to-end learning process based on U-net. Blood vessel segmentation is a pixel-level classification task. Usually, the cross-entropy loss function is used for this kind of segmentation task. But, in the coronary segmentation task, the background occupies a large area of the image, and the blood vessels are unevenly distributed in the image, so dice loss [29] is selected to replace the cross-entropy loss function. If the dice coefficient is higher, the similarity between the predicted result and the ground truth is higher. Also, it is more feasible to train for minimizing the loss value. The loss function is defined as:

2 $$ L_{d i c e}=1-\sum_{m}^{M} \frac{2w_{m} \sum_{i}^{N} p_{(m, i)} g_{(m, i)}}{\sum_{i}^{N} p_{(m, i)}^{2}+\sum_{i}^{N} g_{(m, i)}^{2}}  $$

where *M* is the number of the pixel, the *p*_(*m*,*i*)_ and *g*_(*m*,*i*)_ are predicted probability and ground truth for class *m*. and $\sum _{m}w_{m}=1$ are the class weights, here *w*_*m*_=1/*M*. The final loss function is defined as:

3 $$ L_{l o s s}=L_{d i c e}+L_{r e g}  $$

here *L*_*reg*_ represents the regularization loss [30]. The loss curve is shown in Fig. 5.

### Implementation environment

The model in this method is implemented using PyTorch based on GeForce GTX 1070 GPU. The system is Ubuntu 16.04, and the CPU is intel i5, the RAM is 16G.

### Method results and performance

In this section, we show the results and evaluate the performance of the proposed method on the tasks of coronary angiography video segmentation.

**Fig. 6:**
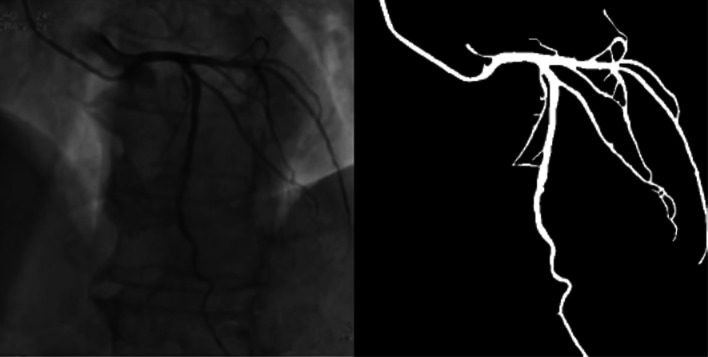
Result of angiography image segementation

**Fig. 7:**
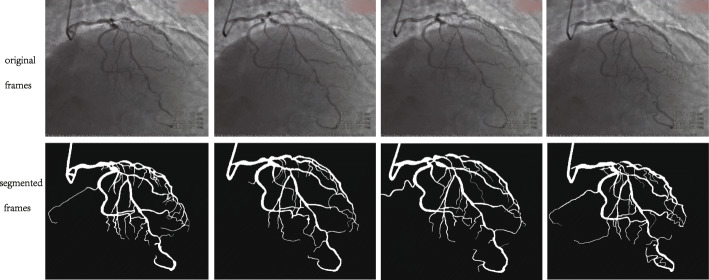
Video sequences segementation results. For frame 3060, 3065, 3070, 3075

Figure 6 shows the segmentation result of one image. Figure 7 shows the results of a video sequence. From the results we can see the detail of the coronary arteries. To evaluate the performance of the vessel segmentation, we compute the sensitivity and the accuracy, which are also calculated in[31].

4 $$ S e n=\frac{T P}{T P+F N}\\  $$

5 $$ A c c=\frac{T P+T N}{T P+T N+F P+F N}\\  $$

where *TP* is the number of true positives, *TN* is the number of true negatives, *FP* and *FN* represent the number of false positives and false negatives. In addition, we also compute the IoU overlap to measure segmentation performance as shown in Fig. 5. Table 1 shows the performance comparison with other method. What needs to be explained here is that we use not only the public data set DRIVE [28] but also our own data set. Our work achieves 0.8739 in *Sen*, 0.9895 in *Acc*, the average values of *TP*, *TN*, *FP*, and *FN* are 3929912.0, 115029685.0, 747732.0, and 860167 for all the test frames. From the performance of the results, the accuracy of our method is significantly improved. The performance benefit from the algorithom architecture in [23] and the enlarged dataset.

**Table 1:** Performace comparison of vessel segmentation

Method	*Sen*	*Acc*
U-Net[19]	0.7537	0.9531
DeepVessel[31]	0.7603	0.9523
Backbone	0.7781	0.9477
Ours	**0.8204**	**0.9867**

## Discussion

**Fig. 8:**
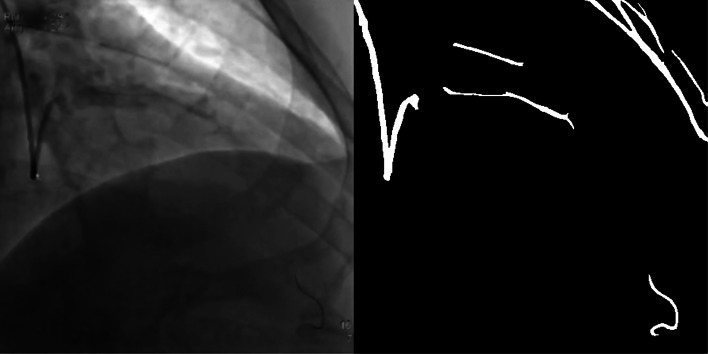
Fail sample

As shown in Fig. 8, two situations can cause segmentation results to fail. First, there are some tissue structures in the video image which have close grayscale value and similar shape with the angiographic vessels, which will cause the tissue to be segmented together. The second is that when the contrast agent is very thin or uneven, the coronary blood vessels will not be segmented. Considering these two situations, we need to make the dataset bigger. At the same time, we need also to consider the differences between the imaging effects of different contrast devices. The time-consuming of this method is about 59ms per frame on a GeForce GTX 1070 GPU, it is almost real-time if the rate set by the coronary angiography system is 15f/s. The algorithm still has room for continuous optimization and will continue to be improved in the future.

## Conclusion

Image segmentation is important in the field of medical image analysis, and segmentation of medical image sequences can obtain more motion information than single-image segmentation, which also has a positive significance for assisting doctors in the diagnosis and treatment. This article introduces the application and implementation of a segmentation method for medical image sequences in coronary angiography video. Compared with previous methods that only focus on single image analysis, we can obtain more coronary information through image sequences. At the same time, we annotated our coronary sequence data set. Experimental results show that this method can segment coronary angiography image sequences with high accuracy. In future work, we will pay more attention to the continuity of information between frames, so that the results look more natural. Our method is now validated on 2D networks and will extend to 3D networks in future work.

## Data Availability

Data related to the current study are available from the corresponding author on reasonable request.

## Abbreviations

Acc: Accuracy
CNN: Convolutional neural networks
CT: Computed tomography
DAC: Dense atrous convolution
DRIVE: Digital retinal images for vessel extraction
FN: False negatives
FP: False positives
Iou: Intersection-over-union
MRI: Magnetic resonance imaging
RMP: Residual multi-kernel pooling
ROI: Region of interest
Sen: Sensitivity
TN: True negatives
TP: True positives
